# Alcohol Consumption and Risk of Gastric Cancer: The Japan Collaborative Cohort Study

**DOI:** 10.2188/jea.JE20190304

**Published:** 2021-01-05

**Authors:** Yuting Li, Ehab S. Eshak, Kokoro Shirai, Keyang Liu, JY Dong, Hiroyasu Iso, Akiko Tamakoshi, the JACC Study Group

**Affiliations:** 1 Public Health, Department of Social Medicine, Osaka University Graduate School of Medicine, Osaka, Japan; 2 Department of Public Health and Community Medicine Department, Faculty of Medicine, Minia University, Minia, Egypt; 3 Department of Public Health, Hokkaido University Graduate School of Medicine, Sapporo, Japan

**Keywords:** gastric cancer, alcohol, JACC study

## Abstract

**Background:**

Alcohol consumption is a potential risk factor for gastric cancer. However, findings from cohort studies that examined the relationship between alcohol consumption and gastric cancer risk among Japanese population are not conclusive.

**Methods:**

A total of 54,682 Japanese men and women participating in the Japan Collaborative Cohort study completed a questionnaire, including alcohol consumption information. The Cox proportional hazard model was used to calculate the hazard ratios (HRs) and 95% confidence intervals (CIs).

**Results:**

After a median 13.4-year follow-up, we documented 801 men and 466 women incident cases of gastric cancer. Alcohol consumption was associated with increased risk of gastric cancer among men (HRs in ex-drinkers and current alcohol consumption of <23 g, 23–<46 g, 46–<69 g, and ≥69 g/d categories versus never drinkers were 1.82; 95% CI, 1.38–2.42, 1.41; 95% CI, 1.10–1.80, 1.47; 95% CI, 1.17–1.85, 1.88; 95% CI, 1.48–2.38, and 1.85; 95% CI, 1.35–2.53, respectively, and that for 10 g increment of alcohol consumption after excluding ex-drinkers was 1.07; 95% CI, 1.04–1.10). The association in men was observed for cardia and non-cardia gastric cancer (HRs in the highest alcohol consumption category versus never drinkers were 9.96; 95% CI, 2.22–44.67 for cardia cancer and 2.40; 95% CI, 1.64–3.52 for non-cardia cancer). However, no such trend was observed in women.

**Conclusions:**

Alcohol consumption is associated with increased risk of gastric cancer among Japanese men, regardless of anatomical subsite of the cancer.

## INTRODUCTION

Gastric cancer is one of the most common cancers in the world, and accounted for 754,000 deaths out of 8.8 million deaths in 2015 according to the World Health Organization estimates.^1^ Although the incidence and mortality rates for gastric cancer have declined worldwide since the middle of the 20^th^ century,^2^ gastric cancer still has high incidence and mortality rates in Eastern Asian countries, where about half of the global gastric cancer load was located.^3^

Several cohort and case-control studies have shown that gastric cancer is associated with *Helicobacter pylori (H.pylori)* infection, lifestyle and dietary factors and genetics. Alcohol consumption was also reported as a potential risk factor for gastric cancer in some previous studies,^4^^,^^5^ but studies on the relationship between alcohol consumption and gastric cancer in the Japanese population, in whom alcohol is commonly consumed by Japanese men, are sparse and the evidence is still not clear, especially from prospective cohort studies.^6^^–^^8^ Results of some meta-analyses showed a significant association of gastric cancer only with heavy alcohol consumption (≥24 g or above per day)^9^^,^^10^; thus, the dose-response relationship between alcohol consumption and gastric cancer could be suggested.

The role of gender differences is still controversial.^11^^–^^14^ Clarifying the association of alcohol consumption with the risk of gastric cancer in men compared with women is particularly important, since the prevalence of alcohol consumption in women is increasing.^15^ Moreover, gastric cancers at cardia (the proximal part of stomach adjoining the esophagus) and non-cardia (the mid and distal stomach) sites may have different etiology.^16^ Therefore, our study aimed to examine the sex-specific dose-response association between alcohol consumption and risk of site-specific gastric cancer among Japanese population.

## MATERIALS AND METHODS

### Study population

The Japan Collaborative Cohort (JACC) Study for Evaluation of Cancer Risks is a large prospective cohort study, the design details and subjects of this study have been described elsewhere.^17^ The JACC study was conducted from 1988 through 1990 and covered a total of 110,585 individuals (46,395 men and 64,190 women) aged 40–79 years in 45 study areas throughout Japan. The baseline information was collected though a self-administered questionnaire covering lifestyles and medical histories. Informed consent was obtained from participants or local community leaders. This study was sponsored by the Ministry of Education, Sport, and Science and approved by the ethics committees of Hokkaido University and Osaka University.

The incidence of cancer was available only in 24 study areas. Of total 65,042 individuals in these 24 areas, we excluded 277 participants with a medical history of gastric cancer and 10,083 participants who missed answers for alcohol consumption (include drinking habit, drinking frequency, and/or drinking amount) at the baseline survey. This left a total of 54,682 participants (22,025 men and 32,657 women) for the analyses.

### Gastric cancer ascertainment

The median follow-up period for cancer incidence surveys was 13.4 years, because the follow-up surveys were discontinued in some study areas before 2009. For analysis, the incidence of gastric cancer was defined as the participants who developed gastric cancer or died of gastric cancer during the observed cancer incidence survey. Because some cases of gastric cancer could not be reported at the time of diagnosis, but rather were reported at the time of death, we counted those cases as incident cases to calculate the incidence. The incidence cancers were based on the records of population-based cancer registries, and the incidence data were coded by the 10th revision of the International Statistical Classification of Diseases and Related Health Problems. Tumors encoded by ICD-10 C16.0–C16.9 were classified as gastric cancers, which were further classified as cardia (ICD-10 codes C16.0), non-cardia (ICD-10 code C16.1–C16.6), and unknown region (ICD-10 codes C16.8–C16.9).

### Baseline survey

A self-administered questionnaire was used to collect the baseline data including information about alcohol consumption, as well as demographic, dietary, and lifestyles characteristics. Subjects were asked if they were never-drinkers, ex-drinkers, or current drinkers to classify their alcohol consumption status. Those who reported being ex-drinkers or current drinkers were also asked about the frequency of drinking per week (less than once/week, 1–2 times/week, 3–4 times week and almost every day), the age at starting drinking, type of alcohol (sake, shochu, beer, wine, or whiskey), and the consumption per occasion in Japanese drinking unit (‘gou’). One (‘gou’) unit is equivalent to 23 g ethanol. We calculated the daily alcohol intake as follows: the weekly alcohol intake frequencies were transformed into a daily drinking score then we multiplied the individuals’ scores by the amount of intake per occasion. In a subsample of 9,732 women and 4,969 men of the JACC study, the serum levels of gamma-glutamyl transferase (GGT) were used to validate the alcohol questionnaire. The sex-specific age-adjusted mean values of GGT were 15 IU/L in women and 20 IU/L in men for never drinkers; respective values were 18 and 26 IU/L for ex-drinkers, 17 and 27 IU/L for current drinkers of <23 g alcohol/d, 25 and 37 IU/L for current drinkers of 23–<46 g/d. In the highest category of alcohol intake in women (≥46 g/d), GGT was 48 IU/L; while in men who drank 46–<69 g/d and ≥69 g/d were 51 and 68 IU/L, respectively.^18^

### Statistical analysis

This study was based on a statistical analysis of the sex-specific incidence of gastric cancer during the follow-up period from 1988–1990 to 2009. Person-years were calculated from the date of completion of the baseline questionnaire to the date of incidence of gastric cancer, death, moving out of the community, or the end of follow up, whichever came first. Baseline characteristics were calculated and presented as mean values (standard deviations) for continuous variable and proportions for categorical variables. We classified alcohol consumption into six categories for men (never-drinker, ex-drinkers, and current drinkers of light-to-heavy alcohol consumption: <23 g/d, 23 to <46 g/d, 46 to <69 g/d, or ≥69 g/d) and five categories for women (never-drinker, ex-drinkers, and current drinkers of light-to-heavy alcohol consumption: <23 g/d, 23 to <46 g/d, or ≥46 g/d). The hazard ratios (HRs) and the corresponding 95% confidence intervals (CIs) were calculated using the Cox proportional hazard model for gastric cancer incidence across alcohol consumption categories. Potential confounding factors for adjustment were baseline age (continuous), family history of parents or siblings with gastric cancer (yes or no), body mass index (<18.5, 18.5–24.9, or ≥25.0 kg/m^2^), education level (primary school, junior high school, high school, or college and higher), stress (too much, much, average, or a little), history of stomach ulcer or duodenal ulcer (yes or no), smoking habits (never smokers, ex-smokers, or current smokers), sport (seldom or never, 1–2, 3–4, and ≥5 h per week), daily walking habits (seldom or never, 1–2, 3–4, and ≥5 h per week), and daily walking habits (seldom or never, <30 min, 30–59 min, or ≥60 min), as well as energy-adjusted intakes of salt, fat, vegetables, and fruits and the total energy intake (sex-specific quintiles). Missing values for these covariates were treated as additional missing categories and their indicators dummy variables were included into the model. The energy-adjusted intakes of selected nutrients were calculated using the residual method.^19^ The reproducibility and validity for dietary intakes of salt, fat, vegetables, and fruits have been reported elsewhere.^20^ *P* for homogeneity was calculated across all the categories (never-drinkers, ex-drinkers, and current drinkers of 0–<23 g, 23–<46 g, 46–<69 g and ≥69 g) using the log-rank test. *P* value for trend across the alcohol consumption categories, excluding ex-drinkers, was calculated using median alcohol consumption in each category. The 10 g increase HR estimation was conducted after excluding ex-drinkers. We tested for possible interaction between alcohol consumption with sex, menopausal status, and smoking status by including cross-product terms of the variables that indicates the categories of alcohol intake multiplied by the variable that indicates the sex (0 and 1), menopausal status (0 and 1), or smoking habit (1 to 3).

In the analyses for the association between alcohol consumption and anatomical subsites of the tumor in men, we considered the highest intake category as ≥46 g/d (combining 46 to <69 g/d and ≥69 g/d categories). A sensitivity analysis was conducted by excluding early incident cases of gastric cancer within the first 5 years of follow up. SAS Version 9.4 software (SAS Institute Inc, Cary, NC, USA) was used in all statistical analyses. Two-tailed *P* values of <0.05 were considered statistically significant.

## RESULTS

At baseline, the respective proportions of never-drinkers, ex-drinkers, and current drinkers were 21.3%, 7.5%, and 71.2% for men and 82.0%, 2.0%, and 16.0% for women, respectively. Compared with never-drinkers, male and female moderate-to-heavy drinkers tended to be younger, to have high perceived mental stress, to be current smokers, and to have lower intakes of fruits and vegetables. In general, history of peptic ulcer was reported among men more than women (Table 1).

**Table 1.** Selected baseline characteristics of participants according to alcohol consumption categories

	Men						Women				

	Never-drinkers	Ex-drinkers	0–<23 g	23–<46 g	46–<69 g	≥69 g	Never-drinkers	Ex-drinkers	0–<23 g	23–<46 g	≥46 g
Number at risk, *n*	4,691	1,662	4,205	5,425	4,263	1,779	26,770	653	4,386	658	190
Ethanol intake, g/d			8.3 (4.6)	26.4 (5.2)	47.0 (3.3)	74.1 (12.0)			5.3 (4.1)	24.2 (3.4)	56.0 (19.2)
Age, years	60 (11)	63 (9)	57 (10)	56 (9)	54 (9)	54 (9)	59 (10)	59 (10)	56 (10)	55 (10)	53 (9)
BMI, kg/m^2^	22.5 (3.0)	22.2 (3.1)	22.7 (2.8)	22.6 (2.7)	22.8 (2.7)	22.9 (2.8)	22.9 (3.1)	23.0 (3.4)	22.9 (3.0)	22.9 (3.1)	23.3 (3.5)
Family history of gastric cancer, %	13	13	12	13	13	13	13	14	13	14	12
History of ulcer, %	22	32	24	23	21	22	11	23	14	15	14
Exercise ≥3 h/wk, %	14	16	15	15	13	12	9	10	12	10	8
Walking ≥30 min/d, %	67	66	67	70	69	69	71	70	73	71	66
College or higher education, %	16	17	21	17	15	14	9	9	12	8	9
High perceived mental stress, %	22	24	25	24	26	30	20	29	24	26	26
Current smokers, %	49	44	45	50	62	69	3	21	7	23	43
**Selected nutrient intakes**											
Energy intake, kcal/d	1,546 (436)	1,466 (431)	1,591 (432)	1,709 (445)	1,838 (436)	2,010 (445)	1,410 (344)	1,303 (339)	1,422 (345)	1,444 (348)	1,609 (387)
Salt intake, g/d	5.5 (1.8)	5.9 (1.8)	5.5 (1.8)	5.4 (1.7)	5.1 (1.7)	4.7 (1.8)	5.0 (1.6)	4.8 (1.5)	4.7 (1.5)	4.4 (1.4)	3.7 (1.6)
Fat intake, g/d	33 (8)	34 (9)	33 (8)	32 (9)	29 (8)	27 (8)	32 (8)	32 (8)	33 (8)	30 (7)	24 (8)
**Food group intakes, g/d**											
Vegetables	250 (301)	275 (311)	245 (298)	238 (301)	219 (299)	187 (293)	298 (321)	303 (319)	308 (325)	302 (330)	256 (323)
Fruit	86 (51)	86 (53)	79 (51)	76 (50)	65 (49)	54 (48)	100 (50)	99 (52)	98 (49)	90 (52)	72 (56)

Means (standard deviations).

Selected nutrient intakes and Food group intakes were energy-adjusted using the residual method.

Among the 54,682 participants (22,025 men and 32,657 women) aged 40–79 years at baseline examination and within the median 13.4-year follow-up period, we identified 1,267 incident cases of gastric cancer (801 in men and 466 in women). Table 2 shows the sex-specific age- and multivariable-adjusted HRs for total gastric cancer among men and women according to alcohol consumption. Ex-drinkers showed a higher risk of gastric cancer compared with never drinkers in both genders (HRs were 1.82; 95% CI, 1.38–2.42 in men and 1.90; 95% CI, 1.15–3.14 in women). The risk showed a dose-response pattern in male current drinkers groups compared with the never-drinker group (HRs were 1.41; 95% CI, 1.10–1.80, 1.47; 95% CI, 1.17–1.85, 1.88; 95% CI, 1.48–2.38 and 1.85; 95% CI, 1.35–2.53; *P*-trend <0.0001 for current drinkers groups of <23 g/d, 23–<46 g, 46–<69 g, and ≥69 g/d, respectively, and that for 10 g increment of alcohol consumption after excluding ex-drinkers was 1.07; 95% CI, 1.04–1.10). No such association was observed in women and the increased risk in female ex-drinker category lost its significance after excluding early gastric cancer cases that occurred within the first 5 years of follow-up (eTable 1); *P*-interaction with sex = 0.076. Stratified analyses by menopausal status did not show significant different results for pre- and post-menopausal women (*P*-interaction >0.1); however, there were a few cases in postmenopausal women at higher alcohol intake categories (data not shown).

**Table 2.** Sex-specific hazard ratios of total gastric cancer according to alcohol consumption categories

	Alcohol consumption								

	Never-drinkers	Ex-drinkers	Current drinkers (per day)				*P* for heterogeneity^d^	*P* for trend^e^	10 g Increment
	
			0–<23 g	23–<46 g	46–<69 g	≥69 g			of alcohol consumption^f^
**Men**									
Number at risk	4,691	1,662	4,205	5,425	4,263	1,779			
Person-years	58,351	17,861	57,077	71,041	58,196	23,716			
Case, *n*	123	84	140	200	186	68			
Age-adjusted HR	1	1.91 (1.44–2.52)	1.40 (1.09–1.78)	1.53 (1.22–1.91)	2.00 (1.59–2.52)	2.00 (1.48–2.70)	<0.0001	<0.0001	1.08 (1.05–1.11)
Multivariable HR (95% CI)^a^	1	1.86 (1.40–2.46)	1.41 (1.11–1.81)	1.47 (1.17–1.84)	1.84 (1.45–2.33)	1.77 (1.30–2.41)	<0.0001	<0.0001	1.06 (1.03–1.10)
Multivariable HR (95% CI)^b^	1	1.82 (1.38–2.42)	1.41 (1.10–1.80)	1.47 (1.17–1.85)	1.88 (1.48–2.38)	1.85 (1.35–2.53)	<0.0001	<0.0001	1.07 (1.04–1.10)

**Women**									
Number at risk	26,770	653	4,386	658	190				
Person-years	350,923	7,612	54,492	7,877	2,339				
Case, *n*	394	17	46	7	2^c^				
Age-adjusted HR	1	1.97 (1.21–3.21)	0.93 (0.68–1.26)	1.00 (0.47–2.12)	1.10 (0.27–4.23)		0	1	0.96 (0.79–1.17)
Multivariable HR (95% CI)^a^	1	1.88 (1.14–3.10)	0.91 (0.67–1.25)	0.98 (0.46–2.09)	1.19 (0.29–4.82)		0	1	0.96 (0.78–1.17)
Multivariable HR (95% CI)^b^	1	1.90 (1.15–3.14)	0.92 (0.67–1.25)	1.03 (0.49–2.20)	1.34 (0.33–5.45)		0	1	0.98 (0.79–1.20)

CI, confidence interval; HR, hazard ratio.

^a^Multivariable adjustment for age, smoking, BMI, family history of gastric cancer, mental stress, education level, history of ulcer, sport, daily walking habits, and total energy (sex-specific quintiles).

^b^Further adjusted for salt, fat, vegetables and fruit intakes (sex-specific quintiles).

^c^Combined the last two alcohol consumption categories.

*P* for sex-interaction in the final adjusted model = 0.076 for gastric cancer.

^d^*P* for heterogeneity was calculated across all the categories (never-drinkers, ex-drinkers and current drinkers of 0–<23 g, 23–<46 g, 46–<69 g and ≥69 g).

^e^*P* for trend was calculated across never-drinker and current drinkers of 0–<23 g, 23–<46 g, 46–<69 g and ≥69 g after excluding ex-drinkers.

^f^10 g increment HR calculation was conducted after the exclusion of ex-drinkers.

The *P* for an interaction term of drinking habit and smoking status was >0.1 in age- and multivariable-adjusted models. However, we presented the stratified analyses for the association between alcohol intake and risk of gastric cancer stratified by smoking status among men in eTable 2. We could not present such stratified analyses for women or for risk assessment by anatomical subsites due to limited number of cases.

Table 3 indicates the result of the association between alcohol consumption and risk of gastric cancer according to its anatomical subsites in men. With reference to never drinkers (HRs for cardia gastric cancer were 5.89; 95% CI, 1.26–27.64, 7.20; 95% CI, 1.62–32.06 and 9.96; 95% CI, 2.22–44.67 in the current alcohol consumption categories of <23 g/d, 23 to <46 g/d, and ≥46 g/d, respectively; *P* for trend = 0.006). The respective HRs for non-cardia gastric cancer were 1.66; 95% CI, 1.10–2.51, 2.02; 95% CI, 1.38–2.95, and 2.40; 95% CI, 1.64–3.52 (*P* for trend <0.001). However, the risk of unknown region gastric cancers was statistically significant in ex-drinkers and highest alcohol consumption category only (HRs were 1.59; 95% CI, 1.11–2.28 and 1.41; 95% CI, 1.05–1.90 respectively).

**Table 3.** Hazard ratios of cardia, non-cardia and unknown regions of gastric cancer according to alcohol consumption categories among men

	Alcohol consumption						

	Never-drinkers	Ex-drinkers	Current drinkers (per day)			*P* for trend^c^	10 g Increment
	
			0–<23 g	23–<46 g	≥46 g		of alcohol consumption^d^
Number at risk	4,691	1,662	4,205	5,425	6,042		
Person-years	58,351	17,861	57,077	71,041	81,911		
							
Cardia							
Cases, *n*	2	7	9	14	16		
Age-adjusted HR	1.00	9.89 (2.05–47.65)	5.42 (1.17–25.14)	6.61 (1.50–29.13)	8.10 (1.80–36.45)	0.007	1.13 (1.00–1.27)
Multivariable HR (95% CI)^a^	1.00	9.11 (1.87–44.38)	5.69 (1.22–26.62)	6.86 (1.54–30.47)	8.31 (1.87–36.93)	0.014	1.13 (1.00–1.27)
Multivariable HR (95% CI)^b^	1.00	8.76 (1.79–42.76)	5.89 (1.26–27.64)	7.20 (1.62–32.06)	9.96 (2.22–44.67)	0.006	1.17 (1.03–1.32)
							
Non-cardia							
Cases, *n*	39	29	58	92	112		
Age-adjusted HR	1.00	2.15 (1.33–3.48)	1.72 (1.15–2.59)	2.13 (1.46–3.10)	2.57 (1.78–3.72)	<0.0001	1.10 (1.05–1.15)
Multivariable HR (95% CI)^a^	1.00	1.93 (1.19–3.14)	1.68 (1.12–2.54)	2.04 (1.40–2.98)	2.39 (1.64–3.49)	<0.0001	1.09 (1.04–1.14)
Multivariable HR (95% CI)^b^	1.00	1.92 (1.18–3.12)	1.66 (1.10–2.51)	2.02 (1.38–2.95)	2.40 (1.64–3.52)	<0.0001	1.09 (1.04–1.15)
							
Unknown region							
Cases, *n*	82	48	73	94	126		
Age-adjusted HR	1.00	1.57 (1.10–2.24)	1.15 (0.84–1.57)	1.12 (0.83–1.50)	1.56 (1.18–2.07)	0.002	1.06 (1.01–1.10)
Multivariable HR (95% CI)^a^	1.00	1.62 (1.13–2.33)	1.18 (0.86–1.62)	1.06 (0.79–1.43)	1.38 (1.03–1.85)	0.070	1.03 (0.99–1.08)
Multivariable HR (95% CI)^b^	1.00	1.59 (1.11–2.28)	1.18 (0.86–1.63)	1.07 (0.79–1.45)	1.41 (1.05–1.90)	0.068	1.04 (0.99–1.08)

CI, confidence interval; HR, hazard ratio.

^a^Multivariable adjustment for age, smoking, BMI, family history of gastric cancer, mental stress, education level, history of ulcer, sport, daily walking habits, and total energy (sex-specific quintiles).

^b^Further adjusted for salt, fat, vegetables and fruit intakes (sex-specific quintiles).

^c^*P* for trend was calculated across never-drinker and current drinkers of 0–<23 g, 23–<46 g, 46–<69 g and ≥69 g after excluding ex-drinkers.

^d^10 g increment HR calculation was conducted after the exclusion of ex-drinkers.

## DISCUSSION

In this prospective study of Japanese men and women, we found that light-to-heavy alcohol consumption was associated with increased risk of gastric cancer morbidity in dose-response fashion among men after adjustment for potential confounding factors. Our result was consistent with the previous findings from case-control and cohort studies of Southern Americans, Europeans, and Asians.^21^^–^^23^ The 2016 World Cancer Research Fund International’s Continuous Update Project report summarized the results from 30 prospective cohort studies and indicated that the consumption of ≥45 g ethanol/day (about 3 drinks a day) was associated with increased risk of gastric cancer (pooled RR 1.06; 95% CI 1.01–1.04). In that report, a 10 g increase in ethanol intake per day was positively associated with risk of gastric cancer in Asians (pooled RR 1.03; 95% CI, 1.01–1.04) more than other ethnicities (pooled RR 1.02; 95% CI, 0.98–1.06 among European and 0.98; 95% CI, 0.87–1.11 among North Americans), and in men (pooled RR 1.03; 95% CI, 1.01–1.05) but not in women (pooled RR 1.02; 95% CI, 0.90–1.15).^24^ The lack of association for women in the present study was consistent with the result of that meta-analysis and could be due to the few cases in the current drinking categories. Moreover, several previous studies have shown alcohol consumption to increase the serum levels of female sex hormones from both ovarian and adrenal sources.^25^ Female sex hormones have been proved to protect against gastric cancer that might partially explain the null association in women.^26^ However, a Swedish prospective study of 61,433 women aged 39–76 years reported that beer consumption but not total alcohol consumption was associated with increased risk of gastric cancer (HR 2.09; 95% CI, 1.11–3.93; *P*-trend = 0.02) for >1 serving of medium-strong (2.8% alcohol)/strong (4.5% alcohol) beer per week versus zero intake, and (HR 1.28; 95% CI, 0.76–2.14; *P*-trend = 0.18) for ≥40 g total alcohol consumption per week versus never drinkers.^13^

A systematic review^8^ of 11 cohort and 11 case-control studies among Japanese indicated that only one Japanese cohort study showed a significant association between alcohol intake and risk of gastric cancer (HR 3.05; 95% CI, 1.35–6.91) for drinkers of ≥50 mL alcohol per day versus non-drinkers,^7^ whereas other cohort and all case-control studies, included in that review, failed to detect any significant association between alcohol drinking and risk of gastric cancer. Shimazu et al attributed the lack of an overall association to the low statistical power in some studies, the misclassifications of ethanol drinking doses or frequencies in some studies that were not detailed in assessing drinking status, and the lack of adjustment for smoking and dietary factors in most of the studies.

Gastric cancers at cardia and non-cardia sites may have different etiology. *H.pylori* infection is a common cause for atrophic gastritis, leading to both of cardia and non-cardia gastric cancers, while cardia gastric cancer is also caused from non-atrophic gastric mucosa, which resembles esophageal cancer.^27^ As for the anatomical site of gastric cancer, three non-Japanese^22^^,^^28^^,^^29^ and three Japanese^30^^–^^32^ previous studies and two meta-analyses^33^^,^^34^ evaluated the association between alcohol consumption and the site-specific risk, but the findings were inconsistent.

Our results match those from the meta-analyses; one meta-analysis based on 17 cohort studies and 58 case-control studies estimated (pooled RR 1.19; 95% CI, 1.01–1.40; *P* = 0.033) for non-cardia and (pooled RR 1.16; 95% CI, 0.98–1.39; *P* = 0.087) for cardia gastric cancers.^33^ Also, in another recent pooled analysis of 20 studies that had 9,669 cases and 25,336 controls, heavy alcohol drinking (>4 drinks/day) compared to never drinking had pooled RRs in both sexes combined of 1.28 (95% CI, 1.13–1.45) for non-cardia gastric cancer and 1.61 (95% CI, 1.11–2.34) for cardia gastric cancer.^34^ However, alcohol intake showed more robust associations with risk of non-cardia gastric cancer in non-Japanese studies. For example, the RR for distal gastric cancer (C16.2–16.6) was 1.3 (95% CI, 1.2–1.5) while that for cardia and upper-third gastric cancer (C16.0–16.1) was 1.3 (95% CI, 0.8–2.2) for ≥25 versus 0 g/d alcohol intake in a cohort study of 669,570 Korean men^22^; the HRs for ≥60 g alcohol/d versus zero intake in a cohort study of 521,457 Europeans men and women were 2.90 (95% CI, 1.53–5.48) for non-cardia and 1.19 (95% CI, 0.56–2.52) for cardia cancers^29^; while in a Chinese case-control study of 1,124 cases and 1,451 controls of both sexes, the respective ORs for heavy alcohol consumption versus never drinking were 1.55 (95% CI, 1.07–2.26) for non-cardia and 0.84 (95% CI, 0.45–1.56) for cardia gastric cancers among men.^28^

On the other hand, the previous Japanese studies showed no association with any gastric cancer site in women, and mixed results for both cardia and non-cardia gastric cancers in men.^30^^–^^32^ Only one case-control study of Japanese men reported a strong positive association of proximal (cardia) gastric cancer for alcohol intake ≥1,350 versus zero alcohol-years (OR 2.46; 95% CI, 1.17–5.17) and middle (non-cardia) gastric cancer (OR 3.29; 95% CI, 1.88–5.76) and distal (non-cardia) gastric cancer (OR 1.56; 95% CI, 0.86–2.84).^32^

We do not have a clear explanation why alcohol consumption in non-Japanese studies was associated with only non-cardia gastric cancer. The different magnitude of association between alcohol consumption and risk of cardia and non-cardia gastric cancers between our study and previous Japanese studies may be attributed partially to the different power to detect significant associations; our study had 2- to 4-fold larger number of gastric cancer cases. Also, the classification of the exposure variable (alcohol intake) differed substantially among the studies; we classified the drinking status as ex-drinkers and three categories of current drinkers compared with never drinkers, while previous studies combined ex- and current drinkers,^30^ or used occasional drinkers 0–3 days/month^31^ or occasional and 0.1–134.9 mL/day^32^ as the reference category.

The biologic mechanisms of alcohol commotion raising risk of gastric cancer are not well understood. However, several mechanisms have been addressed. First, the carcinogenicity of N-nitroso compounds, especially from liquor, is increased by alcohol.^35^^,^^36^ The volatile N-nitroso compound, N-nitrosodime-thylamine (NDMA), in beer, whiskeys, and other hard liquor is a potent carcinogen in animals.^37^ The cohort study of 61,433 Swedish women aged 39–76 years reported an increased risk of gastric cancer associated with high intakes of NDMA calculated from the consumption of each food item (included alcoholic beverages) [HR in the highest (≥0.194 µg/day) vs lowest (<0.041 µg/day) quintiles of NDMA intake was 1.96; 95% CI, 1.08–3.58].^38^ Second, acetaldehyde is the metabolic intermediate of ethanol, which is recognized an animal carcinogen and as a Group 1 carcinogen (sufficient evidence of carcinogenicity in humans) by the International Agency for Research on Cancer.^39^^,^^40^ Alcohol-generated acetaldehyde in the stomach is removed by the aldehyde dehydrogenase 2 (ALDH2) enzyme.^41^ The level of acetaldehyde elimination in the stomach can be affected by the polymorphisms in the genes encoding ALDH2.^42^ ALDH2 Lys allele, which is common in East Asia, is an inactive polymorphism form of ALDH2 and the metabolism of acetaldehyde is significantly reduced.^43^^,^^44^ Alcohol consumption was positively associated with risk of gastric cancer for person with ALDH2 Lys allele, but not for those with ALDH2 Glu/Glu allele according to a recent Japanese case-control study of 1,375 gastric cancer cases and 2,050 controls.^45^ Acetaldehyde can also cause DNA damage in the digestive tract and may have several cancer-promoting effects by causing point mutations, impairing DNA repair, inducing sister chromatid exchanges, inducing metaplasia of epithelium, and forming mutagenic adducts with DNA.^46^

The strengths of our study were its community-based prospective design and the large cohort sample size with high response rate.^17^ Because we evaluated drinking habits before diagnosing gastric cancer, recall errors should not differ between cases and non-cases.

Our study also has several limitations; the main limitation relates to the lack of information on *H.pylori* infection for the total subjects, which is a strong risk factor for gastric cancer.^47^^,^^48^ However, previous Japanese^32^^,^^49^ and non-Japanese studies^29^^,^^50^ reported that alcohol consumption was not correlated with *H.pylori* infection and was independently associated with risk of gastric cancer. Second, although we made adjustments for many potential confounders and stratified by anatomical subsite of the cancer, some confounding and other unmeasured factors, such as a history of gastric surgery and nitrite intake, remain unaccounted for. Third, because of difficulty distinguishing cardia and non-cardia in some cases, some inevitable misclassification could have happened.^31^^,^^51^ Fourth, we had a number of gastric cancer of unknown region (*n* = 11 for overlapping and *n* = 423 for not otherwise specified) and the hazard ratios for that site of gastric cancer cannot be interpreted well.

In conclusion, our findings provide further evidence that alcohol consumption associates with increased risk of gastric cancer among Japanese men, regardless of the anatomical subsite of the cancer.

## APPENDIX A. SUPPLEMENTARY DATA

eTable 1. Sex-specific hazard ratios of total gastric cancer according to alcohol consumption categories after excluding first 5-year gastric cancer cases

eTable 2. Sex-specific hazard ratios of total gastric cancer according to alcohol consumption categories stratified by smoking status among men
