# Supplementary material for: Alcohol Consumption and Risk of Gastric Cancer: The Japan Collaborative Cohort Study
Source: J Epidemiol. 2021 Jan 5;31(1):30–6. doi: 10.2188/jea.JE20190304 (PMC7738647; doi:10.2188/jea.JE20190304)
Supplement: Supplementary file 1 [file je-31-030-s001.pdf]

eTable 1. Sex-specific hazard ratios of total gastric cancer according to alcohol consumption categories after excluding first 5-year gastric cancer cases

	Alcohol consumption							
	Never-drinkers	Ex-drinkers	Current drinkers (per day)				P for trend[d]	10 g increment
			0-＜23g	23-＜46g	46-＜69g	≥69g		of alcohol consumption[e]
Men								
Number at risk	4,647	1,626	4,148	5,344	4,199	1,754		
Person-years	58,236	17,773	56,941	70,846	58,013	23,645		
Case, n	79	48	83	119	122	43		
Age-adjusted HR	1	1.83 (1.28–2.62)	1.20 (0.88–1.64)	1.36 (1.02–1.81)	1.86 (1.40–2.48)	1.78 (1.22–2.60)	<0.0001	1.08 (1.04–1.12)
Multivariable HR (95% CI)[a]	1	1.75 (1.22–2.51)	1.20 (0.88–1.64)	1.29 (0.97–1.72)	1.69 (1.26–2.27)	1.55 (1.05–2.27)	0.001	1.06 (1.02–1.11)
Multivariable HR (95% CI)[b]	1	1.74 (1.21–2.51)	1.21 (0.88–1.65)	1.31 (0.98–1.75)	1.75 (1.30–2.35)	1.63 (1.10–2.41)	0.0004	1.07 (1.03–1.11)
Women								
Number at risk	26,613	645	4,369	657	189			
Person-years	350,525	7,592	54,441	7,874	2,338			
Case, n	237	9	29	6	1[c]			
Age-adjusted HR	1	1.88 (0.97–3.66)	1.03 (0.70–1.52)	1.55 (0.69–3.50)	0.94 (0.13–6.74)		0.476	1.05 (0.85–1.31)
Multivariable HR (95% CI)[a]	1	1.88 (0.95–3.72)	1.01 (0.68–1.50)	1.59 (0.70–3.62)	1.15 (0.16–8.35)		0.424	1.07 (0.85–1.34)
Multivariable HR (95% CI)[b]	1	1.91 (0.97–3.78)	1.02 (0.69–1.51)	1.65 (0.72–3.77)	1.30 (0.18–9.45)		0.346	1.09 (0.87–1.37)

CI, confidence interval; HR, hazard ratio.

[a] Multivariable adjustment for age, smoking, BMI, family history of gastric cancer,mental stress, education level, history of ulcer, sport, daily walking habits, and total energy (sex-specific quintiles).

[b] Further adjusted for salt, fat, vegetables and fruit intakes (sex-specific quintiles).

[c] Combined the last two alcohol consumption categories.

P for sex-interaction in the final adjusted model=0.582 for gastric cancer.

[d] p for trend was calculated across never-drinker and currnet drinkers of 0-＜23g, 23-＜46g, 46-＜69g and ≥69g after excluding ex-drinkers.

[e] 10 g increase HR calculation was conducted after the exclusion of ex-drinkers.

eTable 2. Sex-specific hazard ratios of total gastric cancer according to alcohol consumption categories stratified by smoking status among men

	Alcohol consumption					
	Never-drinkers	Ex-drinkers	Current drinkers (per day)			
			0-＜23g	23-＜46g	≥46g	P for trend[c]
**Never-somkers**						
Number at risk	1,290	248	1,089	1,000	733	
Person-years	16,645	2,832	15,865	14,038	10,509	
Case, n	28	10	28	27	18	
Age-adjusted HR	1	0.92 (0.49–1.72)	0.67 (0.46–0.98)	0.70 (0.48–1.03)	0.67 (0.42–1.07)	0.265
Multivariable HR (95% CI)[a]	1	0.94 (0.50–1.76)	0.68 (0.47–1.00)	0.68 (0.47–1.01)	0.64 (0.40–1.02)	0.434
Multivariable HR (95% CI)[b]	1	0.94 (0.50–1.75)	0.68 (0.46–0.99)	0.68 (0.47–1.01)	0.64 (0.40–1.02)	0.515
**Ex-somkers**						
Number at risk	1,042	639	1,170	1,603	1,400	
Person-years	11,757	6,508	14,703	19,558	18,191	
Case, n	34	31	36	66	61	
Age-adjusted HR	1	1.18 (0.82–1.70)	0.80 (0.57–1.12)	1.10 (0.85–1.41)	1.30 (1.00–1.70)	0.004
Multivariable HR (95% CI)[a]	1	1.21 (0.84–1.74)	0.83 (0.59–1.16)	1.10 (0.85–1.42)	1.25 (0.96–1.63)	0.02
Multivariable HR (95% CI)[b]	1	1.20 (0.83–1.73)	0.83 (0.59–1.16)	1.11 (0.86–1.43)	1.27 (0.98–1.66)	0.02
**Current somkers**						
No. at risk	2,208	704	1,838	2,653	3,741	
Person-years	28,208	7,862	25,072	35,273	50,887	
Case, n	59	38	70	99	169	
Age-adjusted HR	1	1.72 (1.24–2.40)	1.33 (1.03–1.71)	1.27 (1.02–1.58)	1.77 (1.47–2.12)	<0.0001
Multivariable HR (95% CI)[a]	1	1.74 (1.24–2.42)	1.35 (1.05–1.75)	1.25 (1.00–1.56)	1.72 (1.43–2.07)	<0.0001
Multivariable HR (95% CI)[b]	1	1.70 (1.22–2.37)	1.34 (1.04–1.73)	1.25 (1.00–1.55)	1.75 (1.45–2.11)	<0.0001

CI, confidence interval; HR, hazard ratio.

P for interaction in the final fully adjusted model =0.219

[a] Multivariable adjustment for age, BMI, family history of gastric cancer,mental stress, education level, history of ulcer, sport, daily walking habits, and total energy (sex-specific quintiles).

[b] Further adjusted for salt, fat, vegetables and fruit intakes (sex-specific quintiles).

[c] p for trend was calculated across never-drinker and currnet drinkers of 0-＜23g, 23-＜46g, and ≥46g after excluding ex-drinkers.
